# Surgical treatment of large median incisional hernia using the prosthetic mesh introduced behind the rectus abdominis muscle sheath procedure (Rives-Stoppa procedure)

**Published:** 2014-09-25

**Authors:** AG Gangură, RŞ Palade

**Affiliations:** * Surgery Clinic 2, University Emergency Hospital Bucharest; ** Surgery Clinic 1, University Emergency Hospital Bucharest

**Keywords:** Rives-Stoppa procedure, incisional hernia, prosthetic mesh, rectus abdominis muscle sheath

## Abstract

Abstract

A number of 77 large incisional hernias located on the midline of the abdomen were operated following Rives-Stoppa procedure, in a period of five years (2006-2011), in the Surgery Clinic I of the University Hospital Bucharest. The characteristics of the study group were mean age - 62 years, predominance of females - 63 cases (82%), the rate of obesity - 26 observations (34%). Immediate postoperative morbidity was represented by (no. cases): thrombophlebitis (3), prolonged postoperative ileus (6), seroma (12) extended hematic drainage (5), hematoma (4). There were no fatalities. Late postoperative morbidity (no. cases) registered granulomas (4) and recurrence (2). We obtained good and very good results in 71 cases (92%).

## Introduction

Large incisional hernias, upper and/or lower midline, in particular the recurrent ones and the very large and complicated, require a special technique to solve the muscle-aponeurotic defect. 

 From a tactical perspective, three major objectives have been followed: 

 • Making a flexible and resistant parietal structure, free tension sutures, to avoid necrosis, sepsis, dehiscence and subsequent recurrence. 

 • We disagreed with the placement of the prosthesis in a retroperitoneal (in contact with the viscera) manner or on the rectus muscle sheath. 

 • We avoided the replacement of the viscera in the abdominal cavity if it caused high pressure, which could result in serious and immediate functional and breathing problems. 

 We presented our results in treating these patients with synthetic mesh inserted into the rectus abdominis muscle sheath, retromuscular - Rives-Stoppa technique.

## Materials and method 

In a span of five years (2006-2011), 77 incisional hernias, located on the midline, upper and /or lower midline were operated by using the Rives-Stoppa technique. 

 Patient ages averaged for 62 years, with extremes of 24 to 81 years old. 

 Most of the cases were females, with a total of 63 cases (82%) and the male cases were only 14 (18%). 

 The period from the last surgery that caused the incisional hernias or from the last corrective intervention averaged for 15 months, with extremes between 3 and 72 months. 

 A significant percentage (34%), 26 cases were represented by obese patients.

**Table 1:** The number of relapses - corrective intervention by Rives-Stoppa procedure

Number of previous corrective interventions	Number cases	Percentage
0	22	29%
1	38	49%
2	17	22%

 Interventions required general anaesthesia with oro-tracheal intubation, for optimal muscle relaxation and a good anaesthetic comfort. In 60% of the cases, the patients received epidural catheter fitted for postoperative analgesia, which ensured a more efficient ventilation. 

 We administered prophylactic antibiotics during surgery, involving a cephalosporin with an aminoglycoside or beta lactams with an aminoglycoside. 

**Table 2:** Location of the incisional hernias

Musculo-aponeurosis defect location	Number cases(percentage)
supraumbilical	28 (36%)
subumbilical	19 (25%)
over + subumbilical	30 (39%)

 The surgical intervention involved the following major steps: 

 • Iterative incision, with the excision of the old scar. 

 • Dissection of the hernia sac and the aponeurosis surrounding the sac.

 • Incision of the sac and the preservation of most of this tissue.

 • Incision of the lamina rectus abdominis sheaths, and the dissection of the space between the muscle and the posterior lamina of at least 7-8 cm, until the Spiegel line was reached. 

 • This dissection must be performed carefully in order to avoid any injury of the epigastric arteries that can lead to weak postoperative parietal structures. 

 • The haemostasis must be performed very carefully. It can be done by the electrocautery or by the suture, any blood leak will cause hematomas that will compromise the integration of the prosthetic material. 

 • We closed the first layer by suturing the posterior lamina of rectus abdominis muscle. In case this created tension in the abdominal wall, we used part of the peritoneum of the hernia sac that we preserved before.

 • We placed the prosthetic material over this layer, which created the second layer. It was fixed in place by transmuscular sutures at the lateral edge of the rectus abdominis muscle. 

 • We used aspiration drainage with two drain tubes placed retromuscular and externalized through skin incisions that ensured the evacuation of secretions of the virtual spaces. 

 • The third layer was obtained by suturing the anterior lamina of the aponeurosis of rectus abdominis muscle. Relaxation incisions were made on this layer in order to reduce the tension of the suture (nine cases -12%). 

 • The space beneath the skin was also drained with two tubes by separate stab wounds and secured with skin sutures. 

 • In those cases in which there was an excess of fat tissue, we preferred to excise it along with the skin that covered it, for a better result.

**Table 3:** Classification of cases according to the size of the parietal defect, found during

Defect size (cm)	No. cases (%)
10/5	8 (10%)
15/10	31 (40%)
20/10	24 (31%)
25/15	9 (12%)
30/15	5 (7%)

 We practiced the following related surgery: 

 • intraperitoneal dissection, in 54 cases (70%) 

• Partial resection of the omentum, in 23 cases (30%) 

 • Resection of the fat tissue in 28 cases (36%) 

 In most cases, we used polypropylene mesh. 

 The duration of the surgery ranged between 90 and 180 minutes, with an average of 137 minutes. 

 As far as the intraoperative incidents or accidents are concerned, the following should be pointed out: 

 • Damage of epigastric vessels in five cases, which required their ligation. 

 • Damage of the peritoneum sac, when trying to suture it, in three cases. 

 Special attention was given to postoperative care: 

 • The general state of the patient 

 • Surgical wound evolution

 • The drainage quality and quantity

 • Early physical activity

 • Early oral food intake and defecation

 The patient went on antibiotics for 5-7 days postoperatively. 

 Drainage tubes were removed when the drainage volume became insignificant, usually after 7-10 days postoperatively. 

 Results 

 Duration of hospitalization averaged for 12 days.

 Immediate postoperative morbidity consisted of the following: 

 • Seroma -12 cases 

 • Prolonged postoperative ileus -6 cases 

 • Extended drainage -5 cases 

 • Hematoma -4 cases 

 • Thrombophlebitis -3 cases. 

 In the first two to three days after surgery, epidural catheters, used at every few hours or at the request of the patient were very useful in pain management.

 The intensity of controlled pain was moderate to low. 

 There were no immediate postoperative deaths. 

 Late postoperative morbidity was represented by the following: 

 • Granulomas which were treated by excision - 4 cases (5%) 

 • Recurrence of incisional hernia - 2 cases (3%) 

 • Local pain syndrome -2 cases (3%) 

 We achieved good and very good results, both in terms of strengthening the abdominal wall and in aesthetic appearance, in 71 cases (92%). 

## Discussion 

Synthetic prosthesis placement behind the rectus abdominis muscle according to the procedure developed by R. Stoppa [**1**] was addressed to large incisional hernia.

 This technique requires additional measures in order to reduce the risk of infection because: 

 • It involves large dissection areas

 • May lead to haematomas due to bleeding

 • The prosthetic material has appreciable size [**2**]. 

 For these reasons, we avoided the procedure in the following conditions: 

 • Incisional hernias complicated by septic processes (granulomas, abscesses, phlegmons) 

 • Incisional hernias complicated by strangulation. 

 The technique was also avoided in patients with severe cardio-respiratory conditions, due to the risk of worsening them by the following: 

 • The duration of the surgical intervention and of the general anaesthesia. 

 • The dismantling postoperative ventilatory mechanics by increasing pressure on the diaphragm, due to the reintroduction of visceral mass in the abdominal cavity. 

 • Prolonged ileus. 

 Preoperative patient selection especially aimed the following mentioned criteria. We did not use pneumoperitoneum as preoperative preparation method [**3**]. 

 Regarding the technique itself, it was emphasized that it must meet two major objectives (both responsible for any septic complications or relapses): 

 • Avoiding injury to intraperitoneal viscera during dissection. 

 • We should be very careful regarding hemostasis. 

 Polypropylene mesh was especially used in our study for economical reasons. Certainly, there are also superior mesh types: expanded polytetrafluoroethylene (e-PTFE, GORE-TEX, PHOENIX), which confers some advantages such as increased tolerance [**4**], and their use is followed by fewer septic complications [5-7]. 

 Regarding the prosthesis fitting technique, the following should be mentioned: 

 • We did not place prosthesis intraperitoneally, due to the risks of complicated enterocutaneous fistulas [**8**] or its migration in the intestinal lumen [**9**]. 

 • From our experience, more septic complications result after the placing of the prosthesis on the anterior lamina of the rectus sheath. 

 Conclusions 

 • Rives-Stoppa procedure used in large midline incisional hernias was followed by good anatomical, functional and aesthetic result in 92% of the cases. 

 • Good surgical technique, following the principles of haemostasis and infection prevention is necessary to obtain good results.

 • Following these principles and a careful selection of patients will provide a lower rate of morbidity and fewer complications than in the case of other surgical techniques for treatment of incisional hernias.
